# Response to Commentary: Educating the next generation of psychiatrists in the use of clinical neuromodulation therapies: what should all psychiatry residents know?

**DOI:** 10.3389/fpsyt.2024.1474594

**Published:** 2024-09-27

**Authors:** Sahit N. Menon, Tyler Torrico, Bruce Luber, Brian Gindoff, Lisa Cullins, William Regenold, Sarah H. Lisanby

**Affiliations:** ^1^ Noninvasive Neuromodulation Unit, Experimental Therapeutics and Pathophysiology Branch, National Institute of Mental Health, Bethesda, MD, United States; ^2^ Emotion and Development Branch, National Institute of Mental Health, Bethesda, MD, United States

**Keywords:** neuromodulation, medical education, psychiatry training, ECT, TMS, VNS, DBS

A commentary on our article *Educating the next generation of psychiatrists in the use of clinical neuromodulation therapies: what should all psychiatry residents know? Francis A* https://doi.org/10.3389/fpsyt.2024.1436932 raised concerns about expanding neuromodulation training in general psychiatry residency (1).

Firstly, we thank Dr. Francis for highlighting the logistical challenges associated with proposals to enhance general psychiatry residency training in FDA-approved neuromodulation treatments. However, Francis stated that we believe neuromodulation training in general psychiatry residency is preferable to interventional psychiatry fellowships. This is not the position we take in our article (2). Although we advocate for neuromodulation training in general psychiatry residency, we did not state that this should occur instead of subspecialty training following general psychiatry residency training. Our opinion is that all psychiatry residents should have a foundational knowledge of neuromodulation and its clinical indications. Our opinion is consistent with the American Board of Psychiatry and Neurology Psychiatry Core Competencies, which state that psychiatrists “shall demonstrate the following abilities: … To evaluate the indication for, relevance of, and application of the following therapeutic procedures: ECT,… TMS, and VNS.” and “Psychiatrists shall demonstrate knowledge of the following:…patient evaluation and treatment selection, including diagnostic and therapeutic studies, including:…ECT,. TMS, VNS” (3). They are also aligned with ACGME requirements: “residents must demonstrate competence in their knowledge of….indications for and uses of electroconvulsive and neuromodulation therapies; (Core)” (4). Hands-on clinical expertise with neuromodulation does require additional training, and those who desire this experience should pursue the available opportunities, such as interventional psychiatry fellowships.

Second, Francis correctly identifies the opportunity cost associated with expanding training in neuromodulation. However, we believe it is equally important to consider the opportunity cost of not expanding neuromodulation training. As neuromodulation plays a growing role in psychiatric treatment, there is concern that there may be increasing disparities in access to neuromodulation treatments if a broadly based workforce is not properly trained, beginning with foundational didactic milestones during general psychiatry residency. For context, a recent survey of ECT providers found that the proportions of female (29%) and Black or African American (1%) ECT providers were markedly lower than the proportions of female (60%) and Black or African American ECT patients (10%) (3, 5). A diverse workforce, such as psychiatry residents, better equipped to provide and refer patients for neuromodulation treatments enhances equitable access to such care.

Third, Francis raises concern about general psychiatry training programs in the United States that may lack access to clinical sites and clinical preceptors to train residents in neuromodulation. While this is a valid concern, we contend that this lack of access is detrimental not only to the residents in training, but also to the patients they serve who by extension lack access to the full spectrum of psychiatric care.

In summary, we believe that ensuring all residents receive foundational knowledge in neuromodulation will support a future diverse interventional psychiatry workforce and improve patient access to emerging therapies, which is crucial in a rapidly evolving field.
